# Clinical Spectrum of Chikungunya in Pakistan

**DOI:** 10.7759/cureus.1430

**Published:** 2017-07-06

**Authors:** Syeda Naqvi, Shehroz Bashir, Chintan Rupareliya, Abdullah Shams, Pirthvi Raj Giyanwani, Zeeshan Ali, Faiza Qamar, Vijesh Kumar, Vikash Talib

**Affiliations:** 1 Jinnah Postgraduate Medical Centre, Jinnah Sindh Medical University (SMC); 2 Medicine Unit 7, Jinnah Sindh Medical University (SMC); 3 Department of Neurology, University of Missouri, Columbia, Missouri; 4 Internal Medicine, CMH Lahore Medical and Dental College; 5 Civil Hospital Karachi, Dow University of Health Sciences (DUHS), Karachi, Pakistan; 6 Accident & Emergency, Jinnah Sindh Medical University (SMC)

**Keywords:** chikungunya, virus, mosquito, arthralgia, viral fever, fever, purpura, public awareness, analgesics, headache

## Abstract

Background

Chikungunya fever is a pandemic disease caused by an arthropod-borne chikungunya virus (CHIKV). The virus spreads through mosquitoes. This mosquito induced viral illness is clinically suspected on symptoms from fever and severe polyarthralgia. The recent outbreak of chikungunya was reported in November 2016 in the metropolitan city Karachi, Pakistan. We emphasis on the awareness of the etiology and vector control to prevent serious consequences.

Method

A total number of 1275 patients were included in this cross-sectional study. These patients were enrolled based on clinical findings described by Centers for Disease Control and Prevention (CDC). Our exclusion criteria were patients with missing data or having co-infection with dengue or malaria. The patients were tested for chikungunya antibodies, malaria, and dengue. The patients were followed for three months.

Results

Out of 1275 consenting patients from the emergency department, 564 tested positive for chikungunya antibodies and out of these 564 patients 365 had co-infection of dengue and malaria. So based on exclusion criteria, 199 patients had isolated chikungunya infection and were studied for the frequency of clinical symptoms. The most common finding was joint pain and fever on presentation and joint pain was the only chronic finding which persisted.

Conclusion

Our study demonstrated the frequency of clinical findings in chikungunya infection. It also signifies the importance of testing for antibodies because it helped in excluding patients with false positive clinical findings and differentiating co-infection with malaria and dengue. It also gauged patient's view about the cause of this disease.

## Introduction

Chikungunya fever is a viral disease caused by an arthropod-borne virus. Chikungunya is the name derived from a work in non-famous language ‘Kimakonde’. It means “to bend out of shape”, explains the appearance of people suffering from polyarthralgia because of chikungunya virus (CHIKV) fever [1]. It is suspected clinically on symptoms of fever and severe polyarthralgia. These symptoms are sometimes accompanied by other symptoms such as nausea, diarrhea, oral ulcers, headache, myalgia and chest pain. Chikungunya fever is often confused with malaria virus and dengue virus because of confounding effect produced by co-infection and common initial presentation [2-3].

The virus spreads through mosquitoes: Aedes albopictus and Aedes aegypti. These mosquitoes usually bite during daytime and are mostly reported in tropical and subtropical areas [4]. They spread rapidly around the world and there are historical reports of chikungunya fever epidemics in Tanzania during the mid-1950s. Previous outbreaks were reported between 1920 and 1950 in South Asia. The recent epidemic was reported during 2005 in India. Lately, chikungunya outbreak started in November 2016 in Karachi, Pakistan, infecting more than 30,000 inhabitants of Karachi and only 803 cases were reported to World Health Organisation (WHO) since December 2016 [5-7].

The role of antimicrobial agents or vaccination to counter chikungunya fever is under ongoing research [8-9]. Currently, no antiviral treatment targeting the virus is available on the immediate basis. Adequate oral hydration (or intravenous in the event of failure of the oral route) plus non-steroidal anti-inflammatory drugs (NSAIDs) such as Acetaminophen are used to provide symptomatic relief and are the mainstay of the treatment.

## Materials and methods

**Figure 1:**
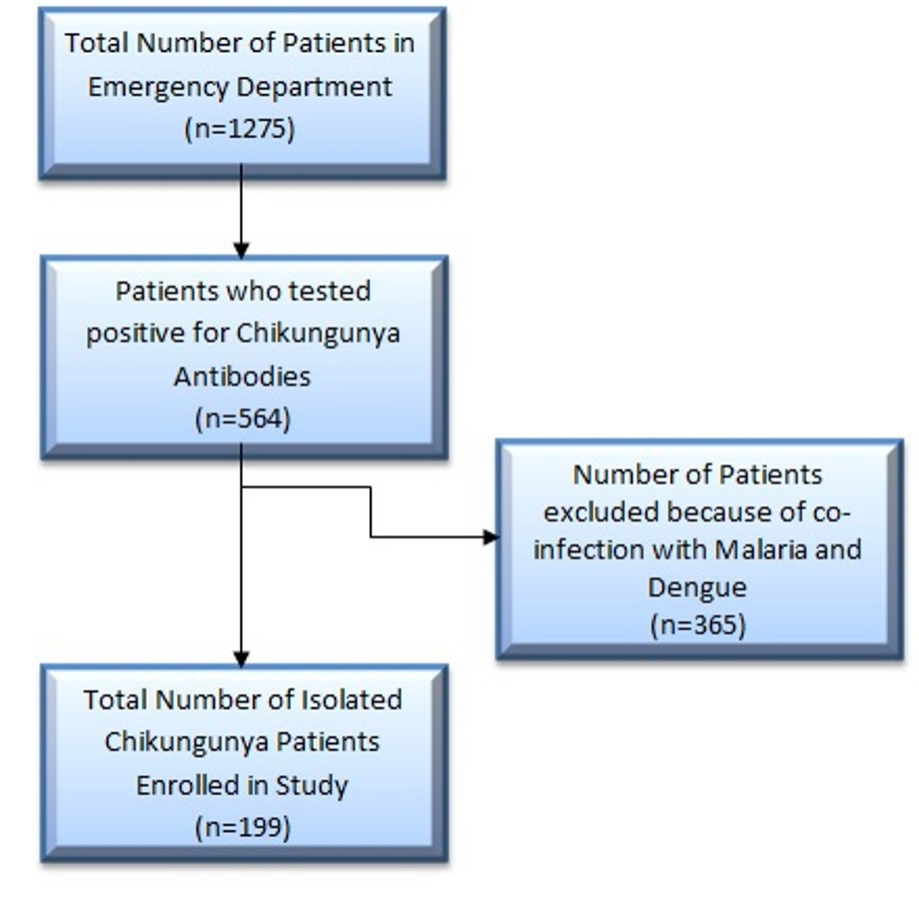
Flow chart depicting selection of the patients

There was a total of 1275 patients in this cross-sectional study. The data was collected from January 2017 to May 2017. We recruited 1275 consenting patients from the emergency department. Out of those 564 patients tested positive for chikungunya antibodies. From 564 patients, 365 patients had co-infection of dengue and malaria. Based on exclusion criteria, 199 subjects had isolated chikungunya infection and were studied for the frequency of clinical symptoms at the time of earliest presentation to the hospital. A detailed illustration is in Figure 1.

The method was explained to the enrolled patients and oral consent was obtained before data collection. These patients were enrolled based on clinical findings described by Centers for Disease Control and Prevention which includes fever more than 101 Fahrenheit, arthralgia, headache, fatigue, nausea, vomiting, rash, oral ulcers, jaundice or bleeding episode. Our exclusion criteria were patients with missing data or having co-infection with dengue or malaria. 

The patients with chikungunya suspected on clinical grounds were tested by convenience blood sampling for chikungunya antibodies, malaria, and dengue. Test for malaria was done through thick and thin films with Giemsa and polymerase chain reaction was used for dengue and chikungunya immunoglobulin G and M (IgG and IgM). The patients were followed over three months time period. 

Statistical analysis was done by Statistical Package for the Social Sciences (SPSS) v 22 (IBM, Armonk, New York, USA), and descriptive statistics were calculated for categorical responses.

## Results

The recent outbreak of chikungunya was reported in the second week of November 2016 in Karachi, one of the densely populated cities of Pakistan. This study took place in emergency departments of four tertiary care hospitals in Karachi. All of them have a huge patient load from almost all four districts of the city.

**Figure 2:**
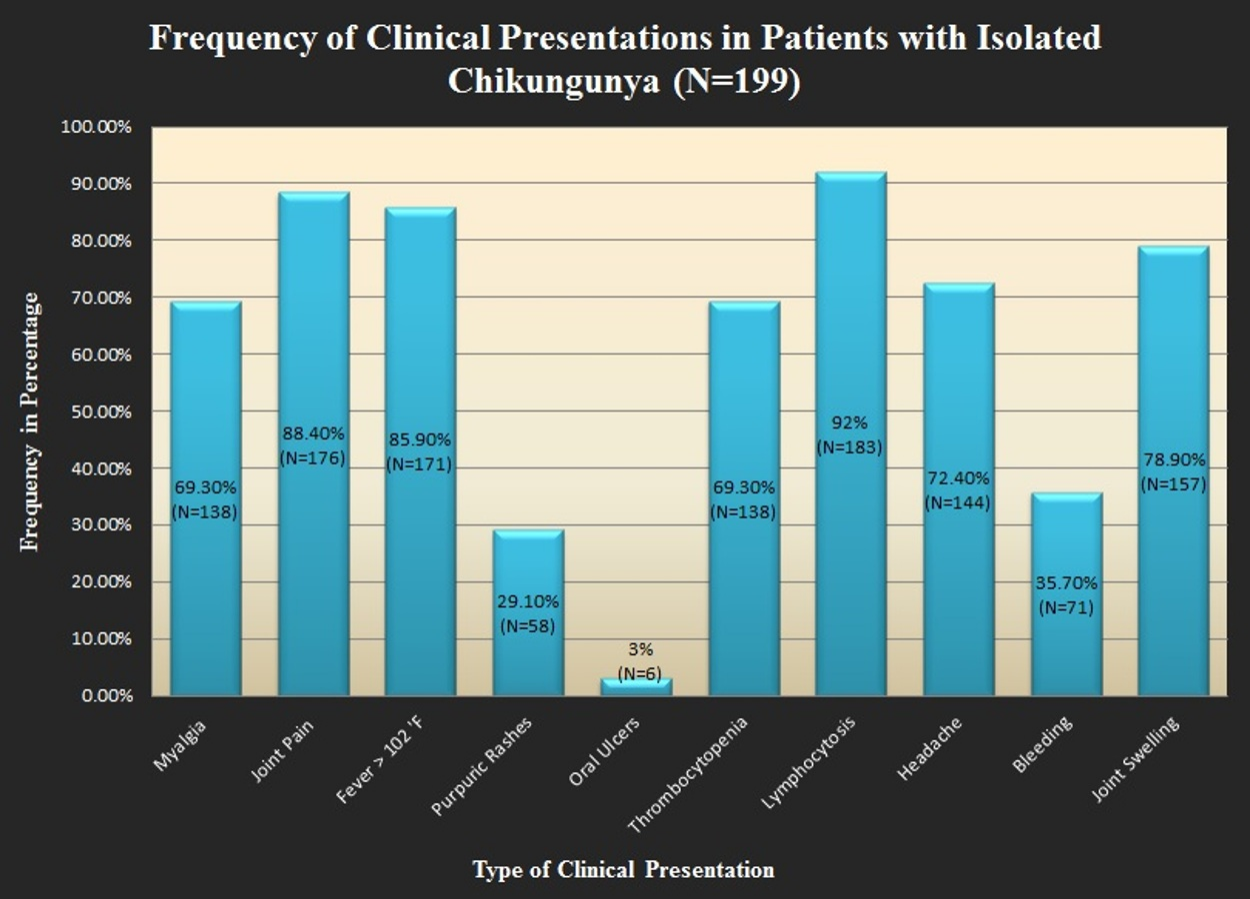
Initial clinical presentation in emergency department

In this study, mean age was 29.52±12.42 years. There were 57.3% males and 42.7% females in this study. Percentages and frequencies were calculated for patient-reported symptoms and clinical findings and they are summarized in Figure 2.

**Figure 3:**
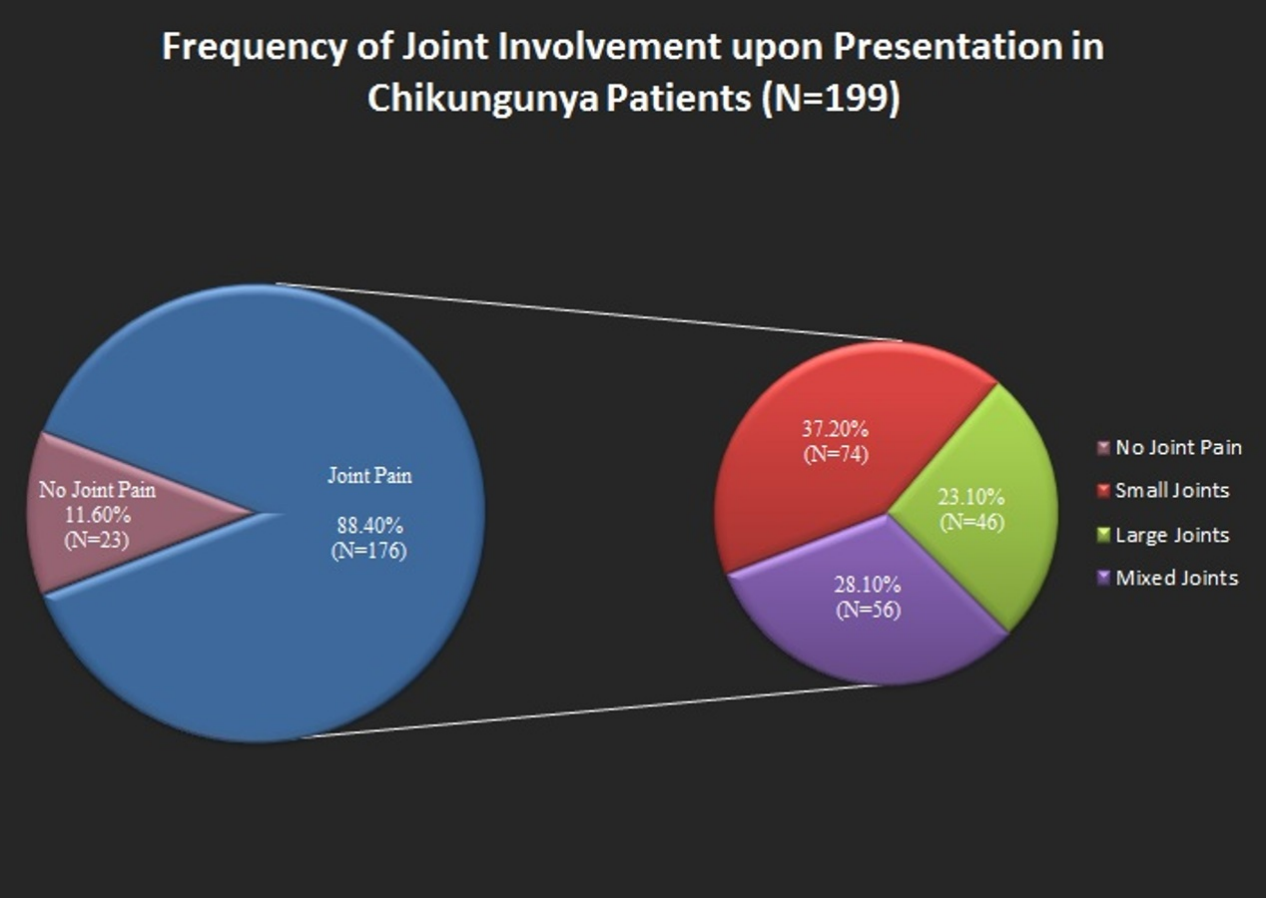
Figure showing the joint involvement

The most common finding was joint pain and fever on presentation. We had formulated questionaries’ pertaining to more details of joint pain. For example; location of joints (proximal vs. distal), the size of joints (small vs. large), functions impaired. Many patients reported mixed joint pains involving both small and large joints. It is shown in Figure 3.

**Figure 4:**
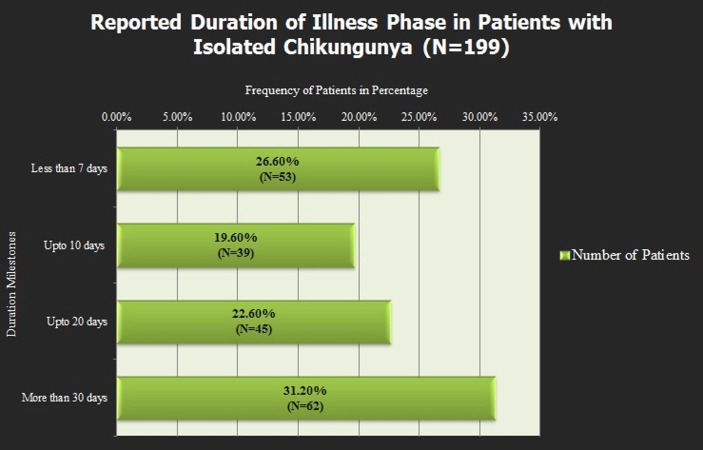
Figure showing the duration of illness

Joint pain was the only chronic finding which persisted. In most of the patients, along with the recovery of clinical findings, laboratory investigations included complete blood count, liver function test, and renal function test was unremarkable but the joint pain persisted. It affects the duration of illness reported by symptoms as shown in Figure 4.

## Discussion

During the last decade, climate change in the form of global warming has hit many areas of the world particularly South Asian countries like Pakistan, Sri Lanka, India, and Nepal. Pakistan, in particular, has witnessed the devastating effect of global warming as the number of catastrophic floods, severe droughts, heat waves and heavy rainfalls have increased lately affecting millions of people. Karachi is the largest and most densely populated city of Pakistan located on the coastline of Arabian Sea with the tropical and humid weather. Substantial climate changes causing summers to be getting harsher and winters to be milder with 15 degrees lowest temperature recorded in December 2016 have provided an excellent medium for nurturing arboviral illnesses specifically dengue, malaria, and chikungunya causing frequent outbreaks in the community [7,10]. Our study aims at exploring the spectrum of chikungunya in Karachi since not only its warm and humid climate but poor sanitary conditions like the lack of waste disposal system, open sewers, and feculent morasses are excellent breeding habitats for mosquitoes that can be found all throughout the city [10-11].

During our five months study, we received 1275 patients on an outpatient basis in four different tertiary hospitals of Karachi who reported classic symptoms of high-grade fever, arthralgia, headache, nausea, vomiting, body aches etc., which are also enumerated by Centre for Disease Control (CDC) [12]. Most of these symptoms are more or less shared by malaria, dengue, and chikungunya. The majority of these patients belonged to low socioeconomic status with poor access to clean housing, education, and proper sanitary measures. Diagnosing infectious disease in resource restricted environment is a big challenge but we focused primarily on diagnosing chikungunya by starting from basic antibody testing for dengue and chikungunya along with thick and thin smear slide test for malaria via Giemsa stain. The PCR test was used to confirm either the diagnosis of dengue or chikungunya or their co-infection. The exclusion criterion was a loss of follow up and co-infection. A total of 199 patients with isolated chikungunya virus was confirmed most of which had arthralgia (88.4%) and high-grade fever (85.9%) as the most common symptoms [13-14].

Chikungunya is mostly self-resolving but its diagnosis should never be undermined since reports of atypical and severe manifestations, sometimes fatal may occur. These include myocarditis, extensive bullous skin lesions, and encephalopathy. Neonatal encephalopathy was also described following vertical transmission at birth. Chronic arthralgia, which may be incapacitating, causes long-term morbidity and have a high economic impact. This may be due to defective clearance and subsequent persistence of chikungunya antigen in macrophages in joints, stimulating ongoing inflammation. Reported rates of chronic arthralgia vary between countries, from 0% to 4.1% at three months in Gabon and Malaysia to 60% at three years in La Reunion, which may reflect differences in underlying population genetics, healthcare practices, or chikungunya strain virulence. The most commonly reported risk factor for chronic arthralgia is older age [15-17].

**Figure 5:**
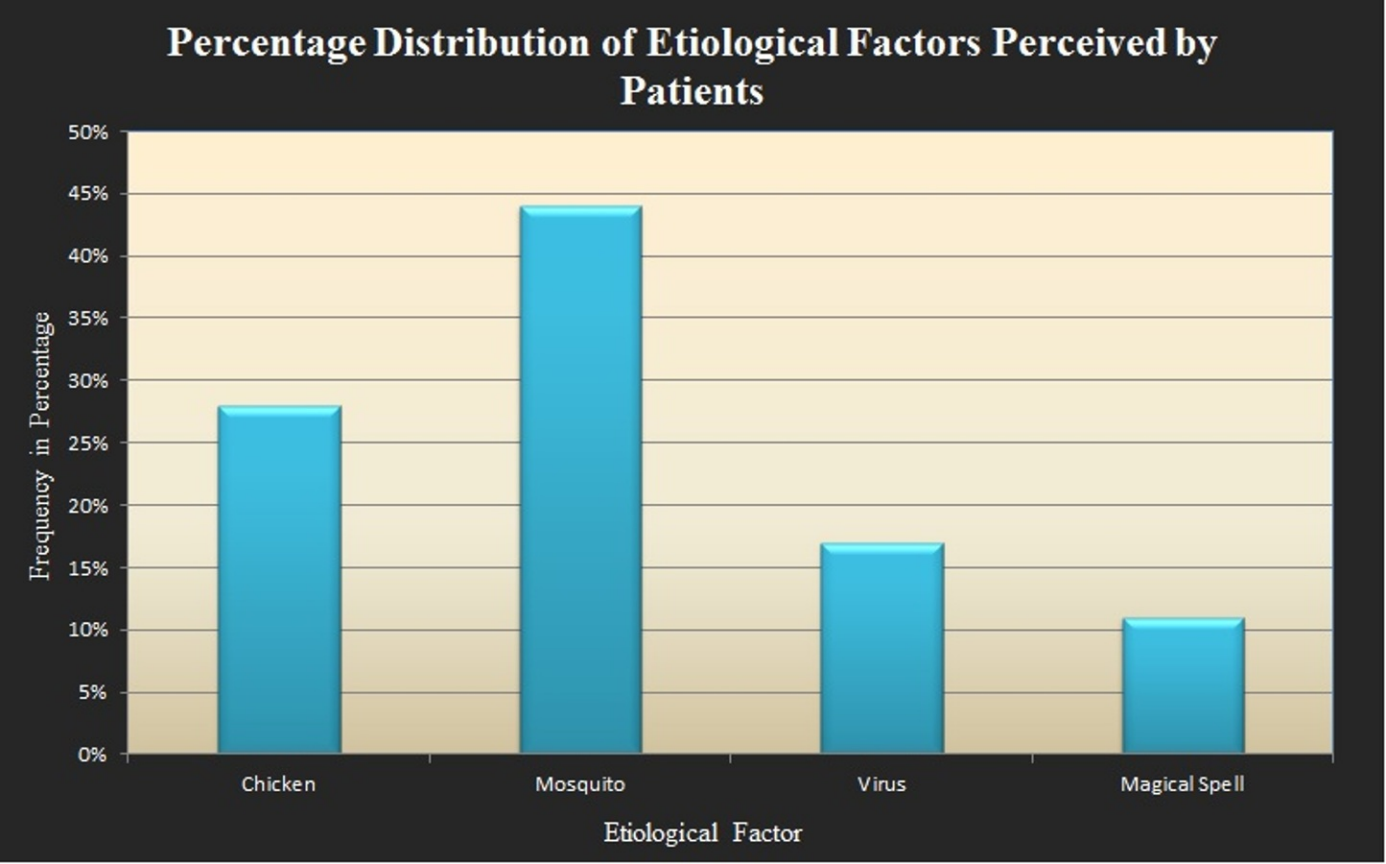
Graphical representation of the public's perception about chikungunya infection

Since the outbreak last year, chikungunya gained widespread popularity due to its fast spread as a mystery illness leading to chaos in the city [7,10]. People had mixed opinion about the disease. This called for the medical community to hold different seminars and campaigns on public awareness. These efforts educated the literate class of the city but underprivileged were still not well educated, which formed the majority. This increased the curiosity and led to this study where we conducted a poll about what is the perspective of the patient about the etiology of this illness. What we found was interesting and it is depicted in this graph (Figure 5).

 

**Figure 6:**
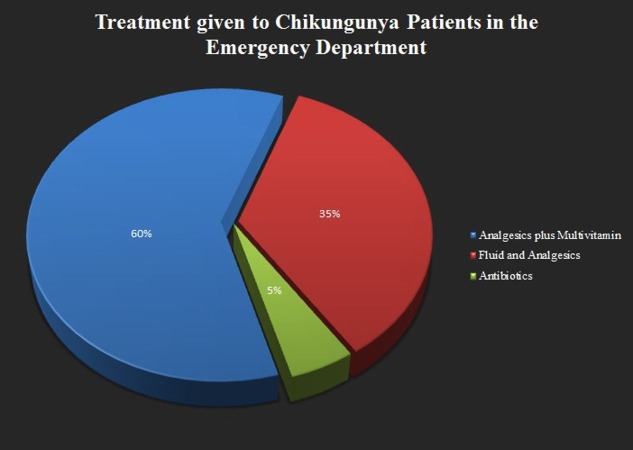
Graphical representation of the treatment given in emergency department

This survey helped us to know different aspects of patient's thinking about the cause of illness which further augmented the other wing of our study regarding the awareness of cause and symptomology of chikungunya among the local masses. Not only the local population, but medical related people were also approached through seminars and symposiums for further improving the efficiency in treating this outbreak. Major of the treatment according to the guideline is based on symptoms which include rest, fluid intake to avoid dehydration, acetaminophens to reduce fever and pain and avoiding aspirin and other nonsteroidal anti-inflammatory drugs (NSAID) (until dengue can be ruled out to reduce bleeding risk) [12,15]. Currently the vaccines and antiviral against chikungunya are under trials and there is no definitive approved vaccine in the market yet [17]. Keeping this in view, the patients in emergency room (ER) were given the following treatment in our study illustrated in Figure 6.

Arboviral illnesses can be prevented by the use of anti-mosquito nets and spray along with full clothing and minimizing the stagnant water sources along with open sewers and also its transmission to areas vulnerable for viral proliferation is of utmost importance in minimizing outbreaks. Chikungunya reached the Western Hemisphere in December 2013 with the first local transmission of the virus on the Caribbean island of St. Martin. The virus has since spread to 44 countries and territories, including the United States, and has affected 1.2 million people. As of February 2015, 47 states have reported a total of 2492 cases. Florida accounts for the largest number of infections, including all 11 known cases of locally-transmitted chikungunya virus infection [13,17].

People suffering from chikungunya virus traveling to vulnerable areas carry the maximum weight in this regard since they are able to transmit the disease during the first week of infection as chikungunya can be found in the blood and passed from an infected person to a mosquito through mosquito bites causing outbreaks. Proper risk factors identified and support from governments for funding awareness campaigns and minimizing the source by vector control can decrease the overall spread of these diseases and outbreaks [18-19].

## Conclusions

This study found that fever and arthralgia along with an increase in lymphocytes during the lab investigation are the commonest manifestations of a virus-induced fever. It also highlights the importance of polymerase chain reaction (PCR) to rule out co-infection with dengue and malaria. Although the treatment is more or less conservative, still any negligence can have dreadful consequences. Regular monitoring of platelet counts and adequate oral hydration are the cornerstones of management. Above all, through this study, we tried to increase awareness about chikungunya and emphasized on the prevention by vector control.
